# On the Complexity Analysis and Visualization of Musical Information

**DOI:** 10.3390/e21070669

**Published:** 2019-07-09

**Authors:** António M. Lopes, J. A. Tenreiro Machado

**Affiliations:** 1 UISPA–LAETA/INEGI, Faculty of Engineering, University of Porto, Rua Dr. Roberto Frias, 4200-465 Porto, Portugal; 2 Institute of Engineering, Polytechnic of Porto, Dept. of Electrical Engineering, R. Dr. António Bernardino de Almeida, 431, 4249-015 Porto, Portugal

**Keywords:** complexity, information theory, multidimensional scaling, music

## Abstract

This paper considers several distinct mathematical and computational tools, namely complexity, dimensionality-reduction, clustering, and visualization techniques, for characterizing music. Digital representations of musical works of four artists are analyzed by means of distinct indices and visualized using the multidimensional scaling technique. The results are then correlated with the artists’ musical production. The patterns found in the data demonstrate the effectiveness of the approach for assessing the complexity of musical information.

## 1. Introduction

The relationships between music and mathematics have been studied for long [1,2]. However, it seems difficult to find a single model for describing a musical work, in spite of it being recognized that we have a glimpse of mathematical structures underneath all types of music [3,4]. A musical work can be represented as a set of one or more time-sequenced digital data streams, reflecting a given time sampling of the original musical source. If a single (`mono’) digital data stream is adopted, then a musical work is represented by a time series (TS), where each sample is a signed floating-point value.

Complexity is one important characteristic of a TS and embeds a description of properties, such as chaoticity, fractality, regularity, and memory [5,6]. In other words, while various properties can describe specific aspects of the TS, the complexity constitutes a general quantitative estimation of their characteristics [6]. Therefore, complexity has become an increasingly prevalent estimator in analyzing TS produced by complex systems, such as in economics [7], finance [8], geo [9], life [10], and social [11] sciences, with the objective of finding the fundamental principles that govern the systems’ behavior [12]. There are no definite guiding rules to the interpretation of the complexity measurements. In general, low complexity indicates that the observed system is more likely to follow some kind of deterministic process that can be finely captured. On the other hand, high complexity represents some data dynamics that are more unpredictable and difficult to understand [6].

A variety of complexity indices has been adopted for tackling art, namely entropy [13,14], Kolmogorov complexity [15,16], fractal dimension [17,18], and others [19,20]. Despite that some of these tools may be correlated, they capture different aspects of the system and, therefore, complement each other [21]. Specifically for the case of music, we can mention the work of Simonton [22] who studied 15,618 themes of classical music and established a connection between melodic complexity and popularity. Eerola and North [23] analyzed the melodic complexity of Beatles’ songs and observed an increasing trend over time. Additionally, they noted some kind of correlation between complexity and the songs’ popularity. Herrera and Streich [24] explored the relationship between the detrended fluctuation analysis exponent and several manually annotated semantic labels of musics. These researchers concluded that there was a link between the exponent and the idea of `danceability’. Li and Sleep [25] investigated the topic of melody classification using a similarity metric based on the Kolmogorov complexity. Ribeiro et al. [26] analyzed 10,000 songs using the complexity–entropy causality plane. The results indicated that this representation could not only discriminate, but also allow a quantitative comparison between songs. Several other works address the use of distinct complexity indices and the adoption of alternative approaches [27,28,29,30].

In this paper we take advantage of the synergies between several tools, namely complexity, dimensionality-reduction, clustering, and visualization techniques, for studying the musical repertories of three singers and one band of different nationalities and ways of being: Frank Sinatra, Rolling Stones, Johnny Hallyday, and Julio Iglesias. In a first phase, the original musical sources are converted into `mono’ digital format and processed using eight distinct complexity indices. Moreover, the results are correlated with the periods of the artists’ careers. In a second phase, a multidimensional scaling (MDS) algorithm is adopted for visualizing complexity. The MDS processes the dissimilarity information calculated with the arc cosine and Canberra distances between the complexity indices, and the loci generated are interpreted under the light of the emerging patterns.

Considering these ideas, Section 2 introduces the mathematical background. Section 3 analyses the musical portfolio of four well-known musicians in the perspective of the eight complexity indices. In addition, the MDS-generated maps are interpreted having in mind the evolution of the artists’ careers. Finally, Section 4 presents the conclusions.

## 2. Mathematical Background

### 2.1. Entropy

The information theory [31,32] has been successfully adopted in the study of complex systems [21,33].

Let us consider a discrete random variable *X* with sample space $\left\{x_{1},\cdots ,x_{i},\cdots ,x_{M}\right\}$ and probability distribution P(X). The Shannon entropy, *H*, of *X* is given by:

(1) $$H\left(X\right)=-\sum_{i=1}^{M}P\left(x_{i}\right)\log P\left(x_{i}\right).$$

The Jensen-Shannon divergence (JSD) measures the dissimilarity between two probability distributions P(X) and P(Y) and is defined as [34]:

(2) $$JSD\left[P(X)\|P(Y)\right]=\frac{1}{2}\left[\sum_{i=1}^{M}P\left(x_{i}\right)\log P\left(x_{i}\right)+\sum_{i=1}^{M}P\left(y_{i}\right)\log P\left(y_{i}\right)\right]-\sum_{i=1}^{M}P\left(z_{i}\right)\log P\left(z_{i}\right),$$

where *X* and *Y* are random variables with sample spaces $\left\{x_{1},\cdots ,x_{i},\cdots ,x_{M}\right\}$ and $\left\{y_{1},\cdots ,y_{i},\cdots ,y_{M}\right\}$, respectively, and $Z=\frac{1}{2}\left(X+Y\right)$.

### 2.2. Permutation Entropy

Different entropy formulations and entropy-based indices have been proposed for data characterization [35,36,37,38,39]. The permutation entropy (PE) was originally proposed to assess the complexity of TS [40]. Let us consider a TS consisting of a series of real-valued samples $\left\{x_{n}:n=1,\cdots ,N\right\}$. We define the parameters d,τ∈N, where the embedding dimension, d≥2, and the embedding delay, τ≥1, represent the length of the TS partitioning sequences and the separation time between their elements, respectively. Let us denote by $\Psi =\{\Pi_{1},\cdots ,\Pi_{d!}\}$ the set of all possible permutations of the ordinals {1,⋯,d}, and by [I] the Iverson bracket [41], such that: $I=\left\{\begin{array}{cc} 1, & \mathrm{if}\;I\;\mathrm{is}\;\mathrm{true}\\ 0, & \mathrm{if}\;I\;\mathrm{is}\;\mathrm{false} \end{array}\right.$.

The procedure for calculating PE can be outlined as follows:

1. For each n=1,⋯,K, with K=N−(d−1)τ,
  1.1 compose the sequence $\left\{x_{n},x_{n+\tau },\cdots ,x_{n+(d-1)\tau }\right\}$;
  1.2 construct the 2×d dimensional array $\left[\begin{array}{cccc} x_{n} & x_{n+\tau } & \cdots & x_{n+(d-1)\tau }\\ 1 & 2 & \cdots & d \end{array}\right]$;
  1.3 sort the array by increasing order of the elements in the first row;
  1.4 denote by $\pi_{n}$ the sequence of numbers in the second row of the sorted array;
2. Compute the probability distribution P(W), where *W* is a random variable with sample space $\left\{w_{1},\cdots ,w_{i},\cdots ,w_{d!}\right\}$ and $P\left(w_{i}\right)=\frac{1}{K}\sum_{n=1}^{K}\left[\pi_{n}=\Pi_{i}\right]$, for i=1,⋯,d!;
3. Calculate PE as
  (3) $$PE=\frac{1}{\log d!}\sum_{i=1}^{d!}-P\left(w_{i}\right)\log P\left(w_{i}\right).$$

The permutation entropy PE lies in the interval 0≤PE≤1. The minimum value PE=0 indicates that the TS is regular, or predictable, while the maximum value PE=1 corresponds to a random TS. The embedding dimension must be chosen such that N≫d! in order to obtain reliable values of PE. For practical purposes, the values d∈{3,⋯,7} and τ=1 are recommended [40].

### 2.3. Statistical Complexity

Another complexity index is the statistical complexity, *C*, given by [42,43]:

(4) $$C=\frac{1}{\kappa }\cdot JSD\left[P(W)\|P(U)\right]\cdot PE,$$

where *U* is a random variable with sample space $\left\{u_{1},\cdots ,u_{i},\cdots ,u_{d!}\right\}$, probability distribution P(U), and $P\left(u_{i}\right)=\frac{1}{d!}$, so that:

(5) $$\kappa =\max_{P}\left\{JSD\left[P(W)\|P(U)\right]\right\}=-\frac{1}{2}\left[\frac{d!+1}{d!}\log\left(d!+1\right)+\log d!-2\log\left(2d!\right)\right]$$

is a normalization constant.

The statistical complexity, *C*, depends on a probability distribution associated with the system, P(W), and on the uniform distribution, P(U). Therefore, for a given PE, there exists a range of possible values of *C*. Indeed, the index *C* provides additional information not captured by the index PE, since it quantifies the existence of correlational structures in the data [42,44].

### 2.4. Kolmogorov Complexity

The Kolmogorov complexity, K(X), of an object X provides a measure of information that is independent of any probabilistic assumptions about the data sequences in X. The measure K(X) is defined as the size of the shortest program that, given an empty object at its input, computes X in a universal computer and then stops [45,46]. The exact value of K(X) is not computable [45,46]. Therefore, approximation schemes are used to obtain its upper bounds, such as the Lempel-Ziv [47], linguistic [48], and compression-based [49] methods.

Lossless compression algorithms approximate K(X) from the size of the compressed object, K(X)≈size[Φ(X)], where Φ(·) denotes the compression algorithm [46]. However, for obtaining a good approximation, the compressor has to be `normal’, meaning that, given X and the concatenation of X with itself, XX, the compressor must generate compressed objects such that size[Φ(X)]≈size[Φ(XX)] [46]. Moreover, for obtaining a complexity index that is independent of size[X] we adopt the complexity ratio, CR, given by:

(6) $$CR=\frac{\mathrm{size}[\Phi (X)]}{\mathrm{size}[X]}.$$

### 2.5. Multidimensional Scaling

Clustering and visualizing data with a large number of attributes is overly important in science [50,51,52,53,54]. The MDS is a computational technique for dimensionality-reduction, clustering, and visualization of multidimensional data [33,55,56,57]. Given a set of objects $x_{i}$, i=1,⋯,L, in a *r*-dimensional space, and a measure of dissimilarity between the pair *i* and *j*, $\delta_{ij}$, the procedure starts by calculating an L×L symmetric matrix, $\Delta =[\delta_{ij}]$, of object-to-object dissimilarities. The matrix Δ is the input to the MDS computational algorithm. In fact, MDS represents objects by means of points located in a *q*-dimensional space (q<r) at distances $\theta_{ij}$. To accomplish this, the MDS iterates multiple configurations and calculates the matrix of distances $\Theta =[\theta_{ij}]$ that minimizes a fitness function. A widely used fitness function is the raw stress:

(7) $$R={\left[\theta_{ij}-f\left(\delta_{ij}\right)\right]}^{2},$$

where f(·) is a linear or non-linear transformation.

The MDS interpretation is based on the patterns of points emerging in the MDS locus. Two similar (dissimilar) objects are shown as two points that are close to (far from) each other. Therefore, we can translate, rotate, and magnify the locus to have a good visualization, because the object-to-object distances remain identical. The MDS axes have neither units, nor special physical meaning.

The MDS quality can be quantified by means of the Shepard and stress plots. The Shepard diagram compares $\theta_{ij}$ and $\delta_{ij}$, for a particular value of *q*. A narrow scattering of the points represents a good fit between $\theta_{ij}$ and $\delta_{ij}$. The stress diagram represents the locus of R versus *q*. Usually, we adopt q=2 or q=3, because such values allow a direct visualization and establish a compromise between achieving low values of R and *q*.

### 2.6. Musical Sounds

In the context of this study, a musical work is a TS, $X=\{x_{n}:i=1,\cdots ,N\}$, representing the arithmetic average of two data streams that result from sampling the original musical source at $F_{s}=44.1$ kHz.

Using the discrete Fourier transform we can express the TS in the frequency domain, resulting in:

(8) $$Y=\left\{y_{k}:k=1,\cdots ,N\right\}=F\left\{X\right\},$$

(9) $$y_{k}=\sum_{n=1}^{N}x_{n}e^{-j\frac{2\pi }{N}\left(k-1\right)\left(n-1\right)},$$

where $j=\sqrt{-1}$ and F{·} is the Fourier operator. Usually, we represent only the first half of the spectrum versus frequency, *f*, or angular frequency, ω=2πf, by considering $k=1,\cdots ,\frac{N}{2}$ and $f=k\frac{F_{s}}{2}/\frac{N}{2}$.

**Figure 1:**
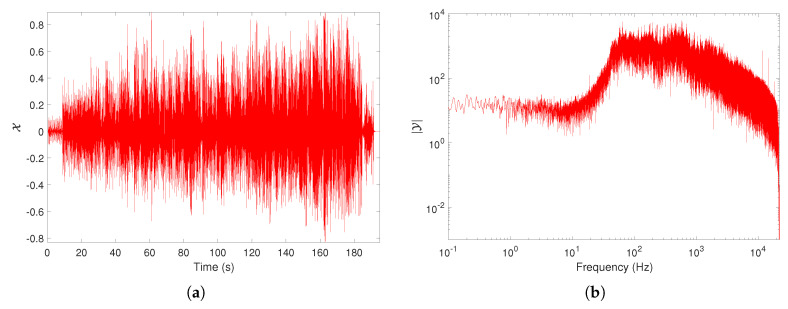
Representation of the the musical work `LA is my lady’ by Frank Sinatra in the time and frequency domains: (**a**) Time series (TS), X; (**b**) amplitude spectrum, |Y|.

The musical sounds have a strong variability, making difficult their quantitative characterization through a single index. Therefore, often several distinct indices are used in the time and frequency domains to capture the rich information embedded in the signal. Figure 1 illustrates the musical work `LA is my lady’ by Frank Sinatra using its TS and amplitude spectrum representations, X and |Y|, respectively.

A variety of features have been proposed for characterizing musical sounds in terms of their dynamics, rhythm, timbre, pitch, and tonality. Music feature extraction involves many signal-processing techniques and forms the basis for many automatic classification algorithms. Several toolboxes are currently available for music and sound feature extraction, such as the MIRtoolbox [58], pyAudioAnalysis [59], and Librosa [60]. The toolboxes often provide not only a set of base features that capture various temporal, spectral, and spectrotemporal properties of the musical signal, but also a considerable number of descriptors derived from the base features by means of descriptive statistics. Typically, all toolboxes provide onset detection, pitch tracking, mel frequency cepstral coefficients (MFCC), chroma, and beat-related features [58,59]. Often, the feature extraction process includes three stages: (i) Dividing each musical work into a set of short-term time windows, or frames, (ii) calculating a collection of features for each frame, and (iii) computing some sort of simple statistics (e.g., mean and variance) of each feature for all frames. The window size is chosen as a compromise between statistical significance and approximate stationarity of the data in each frame. Typical window sizes vary from 20 to 100 ms [59,61,62].

## 3. Complexity Analysis and Visualization

This section addresses the musical repertories of Frank Sinatra, Rolling Stones, Johnny Hallyday, and Julio Iglesias in the perspective of eight complexity indices and the MDS. The musicians were selected for their long and prolific careers, for representing different musical genres, and for singing in different languages.

In a first phase, we apply Equations (1), (3), (4) and (6) to the time and frequency representations of the TS, X and |Y|, respectively. Therefore, we characterize the musical works by means of the set of measures $\left\{H_{T},PE_{T},C_{T},CR_{T},H_{S},PE_{S},C_{S},CR_{S}\right\}$, where the subscripts {T,S} denote the time and spectral complexity indices. For computing $H_{T}$ and $H_{S}$ the probabilities are obtained from the histograms of amplitudes of X and |Y|, respectively, using 100 bins. For the $PE_{T}$, $C_{T}$, $PE_{S}$, and $C_{S}$, we adopt the parameters d=4 and τ=1, that were adjusted by means of numerical experiments. For computing $CR_{T}$ and $CR_{S}$ we adopt the Windows implementation of the gzip compressor, version 1.3.12 (built upon the Lempel-Ziv coding algorithm LZ77). The variability of the individual quantities in the set $\left\{H_{T},PE_{T},C_{T},CR_{T},H_{S},PE_{S},C_{S},CR_{S}\right\}$ is analyzed and correlated with the artists’ musical careers.

In a second phase, we consider that each individual index $\left\{H_{T},PE_{T},C_{T},CR_{T},H_{S},PE_{S},C_{S},CR_{S}\right\}$ captures distinct details of the musical works and that a more complete characterization is accomplished when using all indices simultaneously. However, since an 8-dimensional representation is not feasible, we adopt the MDS technique for dimensionality reduction and visualization.

### 3.1. Frank Sinatra

Frank Sinatra (1915–1998) was one of the most popular singers of the 20th century. Sinatra’s musical style is close to `vocal jazz’, but there is still controversy and debate about this classification. In his artistic career of about 55 years, Sinatra recorded almost 60 studio albums and 300 singles, along with compilations and live albums.

**Figure 2:**
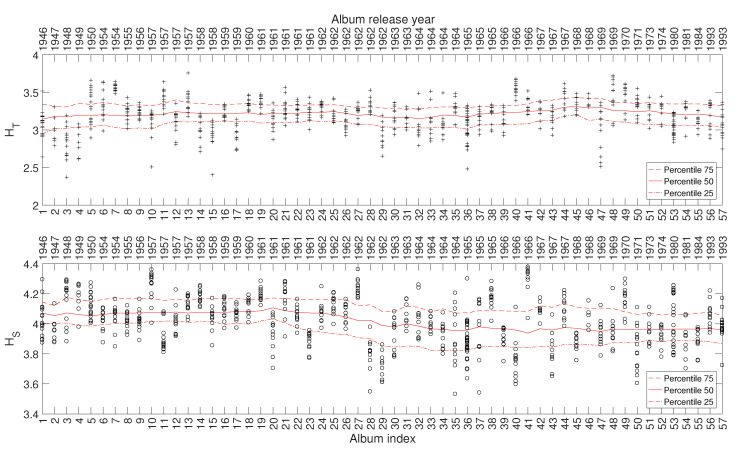
The complexity indices $H_{T}$ and $H_{S}$ versus i=1,⋯,57 for Frank Sinatra’s music.

In this study we consider a total of 707 musical works included in 57 studio albums released in the period 1946–1993. The albums are ordered chronologically and referred to by the sequence i=1,⋯,57. Therefore, we should note that the time lapse between two consecutive values of *i* is not precisely identical. Figure 2 depicts the evolution of the $H_{T}$ and $H_{S}$ (using the black marks + and ∘, respectively) of the musical works versus the index of the album, *i*, where they are included. Given the dispersion of the $H_{T}$ and $H_{S}$ values, we group the musical works in windows of $T_{w}=5$ albums centered at each *i* value (i.e., the window goes from i−2 to i+2), for improving the readability. Then, we calculate the 25, 50, and 75 percentiles, and represent the results by means of three continuous lines. Numerical experiments showed that this width establishes a good compromise between limited volatility and accurate discrimination. Lower values of $T_{w}$ increase the detail, but blur the charts, while higher values of $T_{w}$ tend to filter too much the time details. We verify that there exist relationships between the evolution of $H_{T}$ and $H_{S}$ and the different periods of Sinatra’s artistic career, even knowing that these periods are neither rigidly defined nor absolutely consensual. For the other complexity indices, we reach similar results and, therefore, their representation is omitted here for the sake of parsimony.

The MDS is adopted for reducing dimensionality from an 8- to a 3-dimensional space, allowing a direct interpretation of the results. We start by constructing a 57×8 dimensional array, $W=[w_{ik}]$, where $w_{ik}$, i=1,⋯,57, k=1,⋯,8, represents the median of the *k*th complexity index when grouping the musical works into windows of $T_{w}=5$ albums centered at each *i* value. Then, we calculate the dissimilarity matrices $\Delta_{A}=\left[\delta_{A}\left(u_{i},u_{j}\right)\right]$ and $\Delta_{C}=\left[\delta_{C}\left(u_{i},u_{j}\right)\right]$, {i,j}=1,⋯,57, where $\delta_{A}$ and $\delta_{C}$ denote the arc cosine and Canberra distances between $u_{i}=\left[w_{ik}:k=1,\cdots ,8\right]$ and $u_{j}=\left[w_{jk}:k=1,\cdots ,8\right]$, respectively. The two distances are given by:

(10) $$\delta_{A}\left(u_{i},u_{j}\right)=\arccos\left(\frac{\sum_{k=1}^{57}u_{ik}u_{jk}}{\sqrt{\sum_{k=1}^{57}u_{ik}^{2}}\sqrt{\sum_{k=1}^{57}u_{jk}^{2}}}\right),$$

(11) $$\delta_{C}\left(u_{i},u_{j}\right)=\sum_{k=1}^{57}\frac{|u_{ik}-u_{jk}|}{|u_{ik}\left|+\right|u_{jk}|}.$$

Other distances can be adopted, but several numerical experiments with distinct alternatives [63] confirmed that the arc cosine and the Canberra distances yield good results. Each of the matrices $\Delta_{A}$ and $\Delta_{C}$ is processed by means of the MDS for constructing the loci of objects that represent the evolution of complexity.

**Figure 3:**
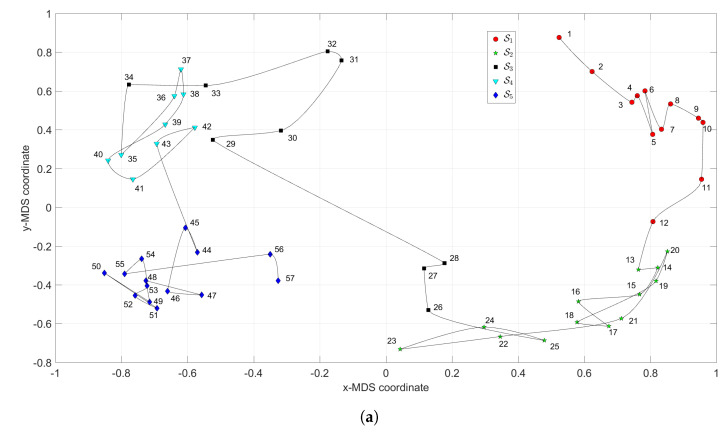

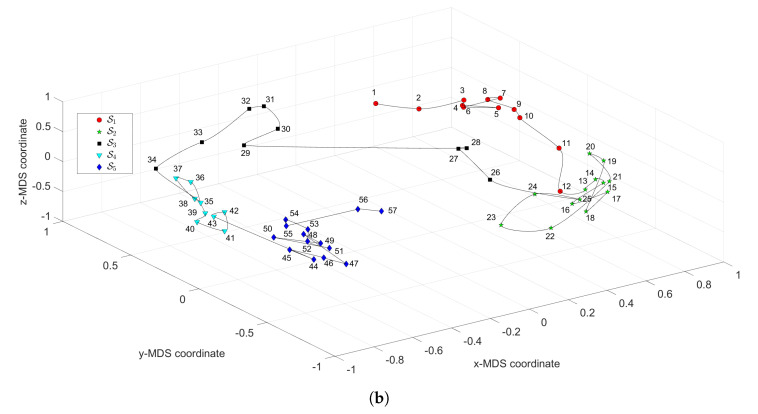
The multidimensional scaling (MDS) maps for Sinatra’s music with $\Delta_{A}$ and i=1,⋯,57: (**a**) q=2; (**b**) q=3.

**Figure 4:**
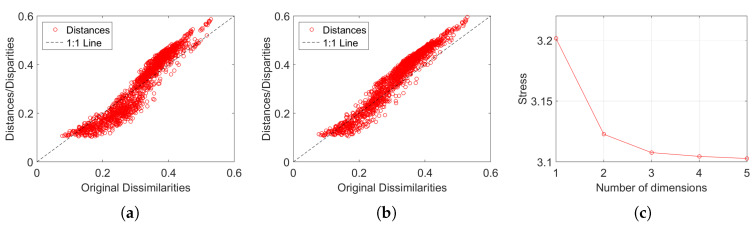
The MDS assessment charts for Sinatra’s music, with $\Delta_{A}$ and i=1,⋯,57: (**a**) Sheppard for q=2; (**b**) Sheppard for q=3; (**c**) stress versus q=1,⋯,5.

**Figure 5:**
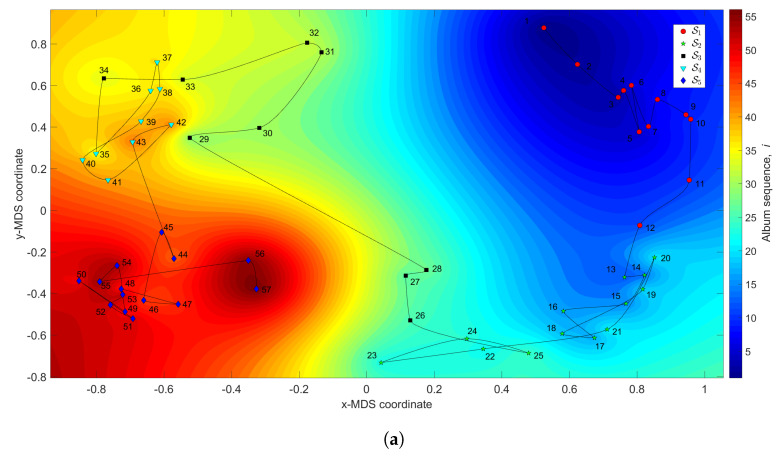

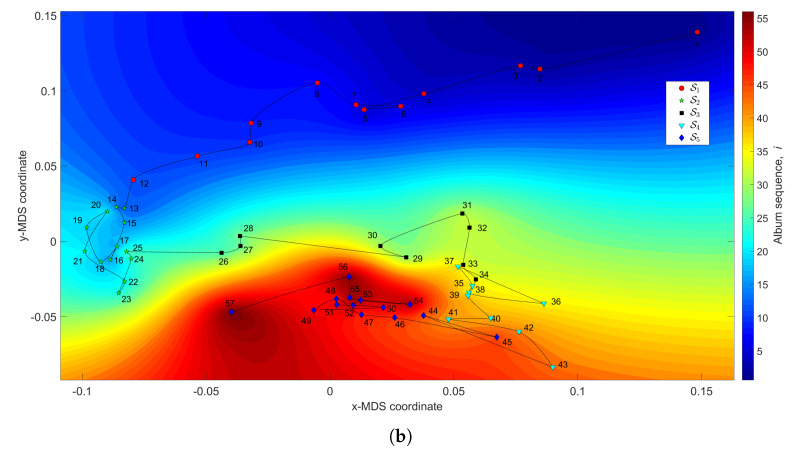
The MDS maps for Sinatra’s music. The (x,y) coordinates are generated by means of the MDS with q=2 and the matrices: (**a**) $\Delta_{A}$; (**b**) $\Delta_{C}$. The *z* coordinate represents the albums’ sequence, *i*, interpolated with radial basis interpolation (RBI).

Figure 3 depicts the MDS maps for Sinatra’s music, for q=2 and q=3, with $\Delta_{A}$ and i=1,⋯,57. Figure 4a,b shows the corresponding MDS assessment charts. The Shepard diagram reveals a small scatter around the 45 degree line, demonstrating that there exists a good fit between the original and the reproduced distances. The stress plot shows that the maximum curvature of the line occurs close to q=2. Therefore, we conclude that q=2 yields a good compromise between accuracy and readability of the locus of points, while q=3 just leads to a marginal improvement, since the *z*-MDS coordinate carries reduced additional information. Alternatively, for taking advantage of present day computational visualization, we adopt a distinct 3-dimensional representation, with q=2 and the *z* coordinate of the map representing the albums’ sequence, *i*, interpolated with radial basis interpolation (RBI) [64] at each point with coordinates (x,y) produced by the MDS. The thin-plate spline $\phi \left(\epsilon \right)=\epsilon^{2}\log\epsilon$ RBI function is considered, where the variable ϵ denotes the Euclidean distance between the points generated by the MDS for q=2 and points in the xy MDS plane. Figure 5a,b depicts the results obtained for $\Delta_{A}$ and $\Delta_{C}$, respectively. The Shepard and stress diagrams are omitted here, since they are of the same type as the ones presented in Figure 4.

We verify the emergence of five clusters, $S_{r}$, r=1,⋯,5. In the first, $S_{1}$ (i.e., albums 1≤i≤12), the complexity varies strongly, meaning that the characteristics of the musical works evolved considerably. This cluster corresponds to albums released in the years 1946–1957. For $S_{2}$ (13≤i≤25), the complexity has limited evolution and corresponds to albums recorded during the years 1957–1962. In the cluster $S_{3}$ (26≤i≤34) the trajectory changes direction and has another large excursion, corresponding to musical albums recorded in the years 1962–1964. The cluster $S_{4}$ (35≤i≤43) includes albums from 1964 up to 1967 and we verify that the complexity has a limited evolution. Finally, for the cluster $S_{5}$ (44≤i≤57), another route occurs, smaller than the previous ones for $S_{1}$ and $S_{3}$. Here, the complexity evolves slowly until the two last albums, consisting of duets (`Duets I and II’), which explains the variation at the end of the career. It is also interesting to see that between two consecutive clusters $S_{i}$ and $S_{i+1}$, (i=2,3,4), we have always a trajectory tangle revealing the artist’s search for the new direction of work.

We now analyze the musical repertory of Frank Sinatra by means of classical musical features, instead of general complexity indices. Therefore, each of Sinatra’s musical works (707 in total) is split into 50 ms non-overlapping frames, and a collection of 34 features is extracted for each time frame. After, for each feature, the average, the standard deviation, and the ratio between the average and the standard deviation are computed. Thus, each piece of music is characterized by a 34×3 dimensional vector, $f_{i}$. Herein, we adopt the zero crossing rate, energy, energy entropy, spectral centroid, spectral spread, spectral entropy, spectral flux, spectral rolloff, mel frequency cepstral coefficients (13 values in total), chroma vector (12 values in total), and chroma deviation. For a detailed description about these features, interested readers can refer to [59]. It should be noted that a different set of features could have been used, since others are also available, and consequently many combinations are possible.

**Figure 6:**
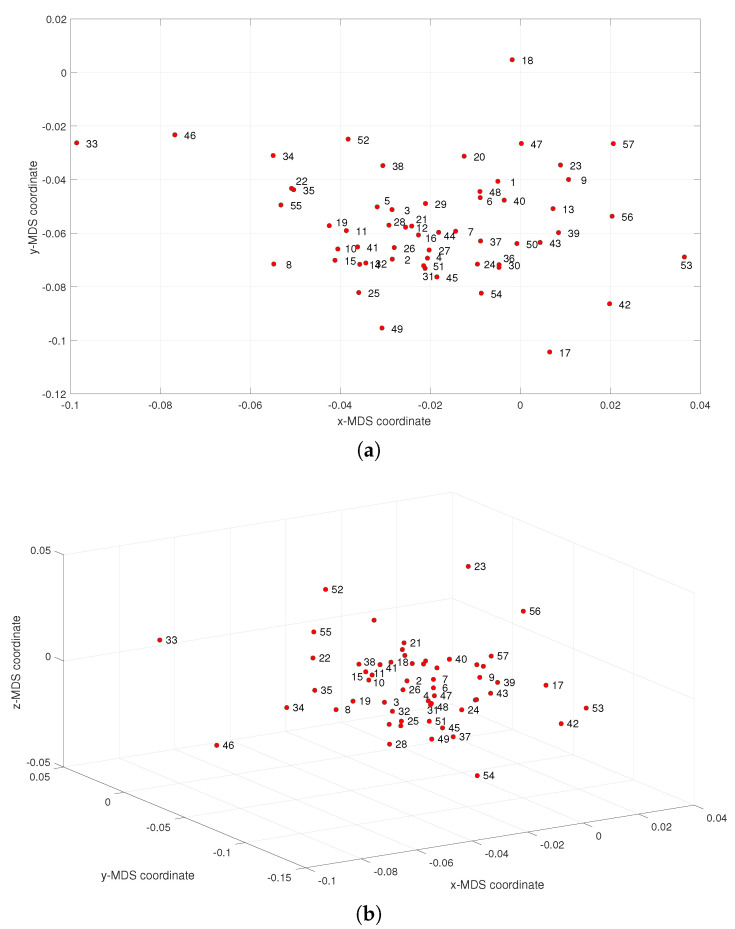
The MDS maps for Sinatra’s music obtained the classical musical features and $\Delta_{A}^{†}$: (**a**) q=2; (**b**) q=3.

We compute the 707×707 dimensional matrix $\Delta_{A}^{†}=\left[\delta_{A}^{†}\left(f_{i},f_{j}\right)\right]$, where $\delta_{A}^{†}\left(f_{i},f_{j}\right)$ denotes the arc cosine distance between the feature vectors $f_{i}$ and $f_{j}$, i,j=1,⋯,707. The matrix $\Delta_{A}^{†}$ is used as the input to the MDS. Since the MDS technique outputs a large number of points, we post-process the results by (i) grouping the musical works into windows of $T_{w}=5$ albums centered at each *i* value, and (ii) calculating the medians of the corresponding (x,y,z) MDS coordinates. Figure 6 depicts the resulting 57-point 2- and 3-dimensional maps. Contrary to the previous experiments, in Figure 3, we do not see the emergence of any pattern. This means that in the perspective of this study, general complexity measures unravel characteristics somehow overlooked by specialized feature descriptors. While a systematic comparison of the two possible strategies, that is, the balancing between general indices and specialized ones is of interest, hereafter we follow the first due to its superior performance in the present case.

### 3.2. Rolling Stones

The English rock band Rolling Stones was created in 1962. The original band included the vocalist Mick Jagger, the guitarists Keith Richards and Brian Jones, the bassist Bill Wyman, the drummer Charlie Watts, and the keyboardist Ian Stewart. Ian Stewart left the group in 1963 and Brian Jones in 1969, being replaced by Mick Taylor, who remained until 1974. In 1975, the guitarist Ron Wood joined the band. The Rolling Stones quickly became the `bad-boys’ band, with an image of sex, drugs, and rebelliousness, in contrast to their contemporary band `The Beatles’. Their music was influenced by different styles from blues and jazz to dance and early rock-and-roll. The Rolling Stones are one of the most successful and acclaimed rock bands of all time. For more than 50 years, they released about 30 studio albums along with several live albums and compilations.

In the sequel we consider a total of 317 musical works included in 27 studio albums released in the period 1964–2005.

**Figure 7:**
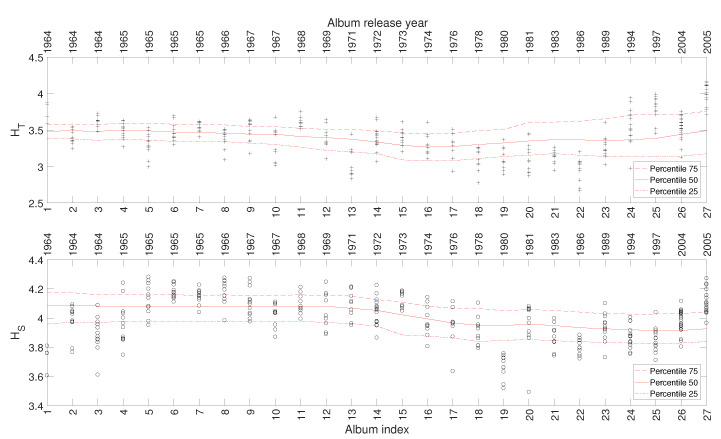
The complexity indices $H_{T}$ and $H_{S}$ versus i=1,⋯,27 for the Rolling Stones’ music.

Figure 7 depicts the 25, 50, and 75 percentiles of $H_{T}$ and $H_{S}$ versus i=1,⋯,27, calculated as explained in the previous subsection. Again, we verify a relationship between the evolution of the indices and the different periods of the band’s artistic career. For the other complexity indices, we reach to similar results and, therefore, their representation is omitted here.

**Figure 8:**
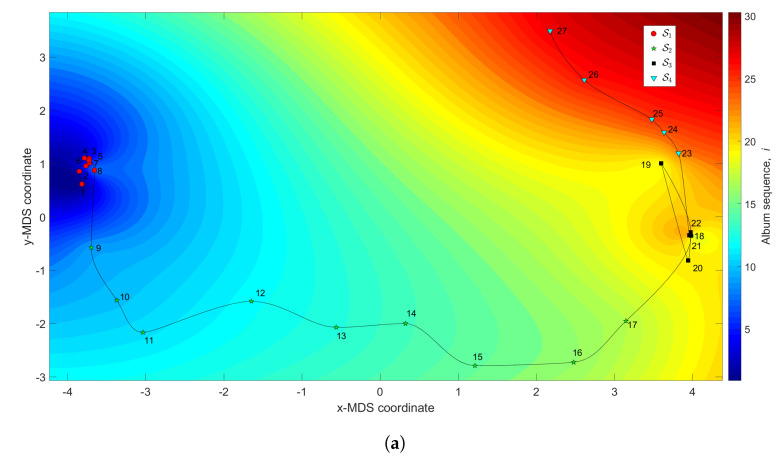

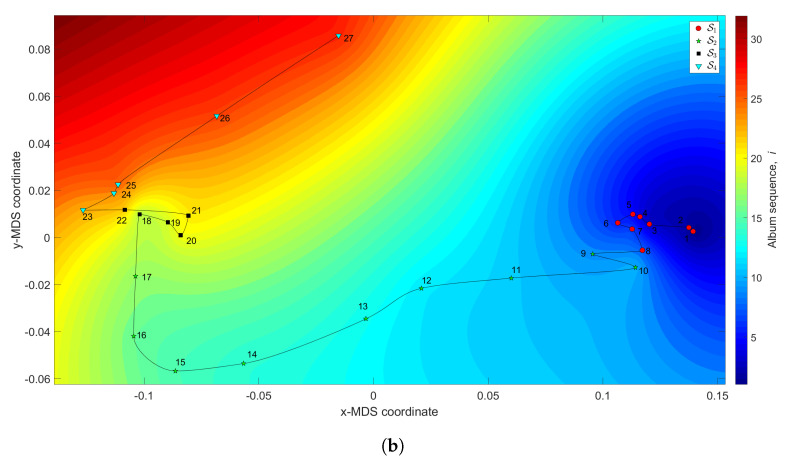
The MDS maps for the Rolling Stones’ music. The (x,y) coordinates are generated by means of the MDS, with q=2 and the matrices: (**a**) $\Delta_{A}$; (**b**) $\Delta_{C}$. The *z* coordinate represents the albums’ sequence, *i*, interpolated with RBI.

Figure 8 depicts the 3-dimensional map of the Rolling Stones’ career. The (x,y) coordinates are obtained by the MDS with q=2 and the dissimilarity matrices $\Delta_{A}$ and $\Delta_{C}$, while the *z* coordinate addresses the albums’ sequence, *i*, interpolated with RBI. The Shepard and stress diagrams are not represented, since they are of the same type as the ones presented in Figure 4.

We verify that the complexity loci have two small tangles, $S_{1}$ (1≤i≤8) and $S_{3}$ (18≤i≤22), corresponding to albums released between the years 1964 and 1966, and 1978 and 1986, respectively. These tangles intermediate two large excursions, $S_{2}$ (9≤i≤17) and $S_{4}$ (23≤i≤27), that include the albums released during the periods 1967–1976 and 1989–2005, respectively. In one hand, we can notice that the periods of complexity stagnation, $S_{1}$ and $S_{3}$, comprise the early discography and the commercial success peak periods. On the other hand, the periods of strong complexity variation, $S_{2}$ and $S_{4}$, include some troubled years and the entry of Ronnie Wood to the band, and the comeback and record-breaking tours that took place after the near break up.

### 3.3. Johnny Hallyday

Johnny Hallyday (1943–2017) was a French singer, songwriter, musician, and actor. He is considered the father of French rock and roll and sometimes he is referred to as the French Elvis Presley. Johnny’s artistic career lasted about 55 years and had plenty of musical success, especially in France and French-speaking countries. He recorded about 50 studio albums, as well as diverse compilations. He is well remembered for his spectacular live concerts with some shot of eccentricity.

**Figure 9:**
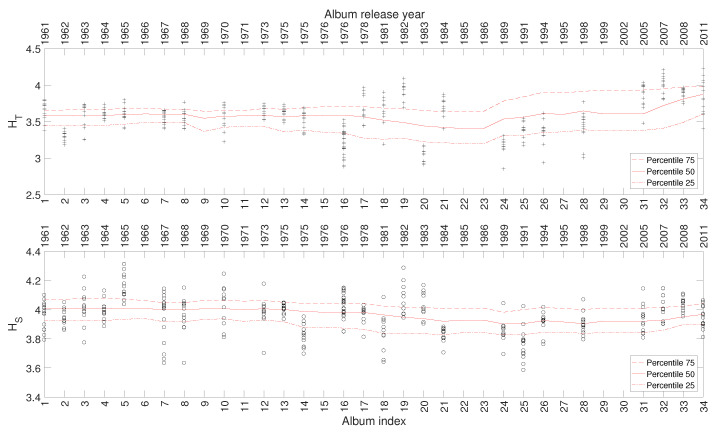
The complexity indices $H_{T}$ and $H_{S}$ versus i=1,⋯,34 for Johnny Hallyday’s music.

Figure 9 depicts the 25, 50, and 75 percentiles of $H_{T}$ and $H_{S}$ versus i=1,⋯,34, calculated for a total of 325 musical works included in 34 studio albums released in the period 1961–2011. As mentioned for the previous artists, a relationship emerges between the complexity indices and the evolution of Johnny Hallyday’s career.

**Figure 10:**
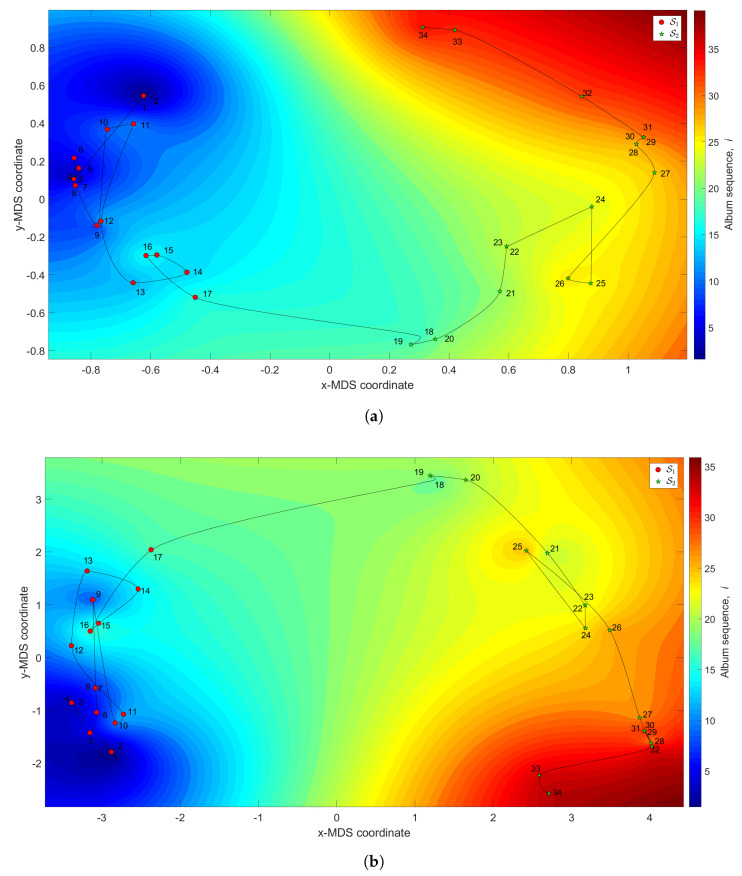
The MDS maps for Johnny Hallyday’s music. The (x,y) coordinates are generated by means of the MDS with q=2 and the matrices: (**a**) $\Delta_{A}$; (**b**) $\Delta_{C}$. The *z* coordinate represents the albums’ sequence, *i*, interpolated with RBI.

Figure 10 represents the 3-dimensional map where the (x,y) coordinates are generated by the MDS with q=2 and the dissimilarity matrices $\Delta_{A}$ and $\Delta_{C}$, and the *z* coordinate denotes the albums’ sequence, *i*, interpolated with RBI.

We observe the emergence of two main clusters. The first is a large tangle, $S_{1}$ (1≤i≤17), corresponding to albums released between the years 1961 and 1978. In this period, Hallyday recorded several French versions of American hits and French songs. The second, $S_{2}$ (18≤i≤34), includes albums between the years 1981 and 2011. It begins in the early 1980s, when Johnny’s career seemed to be on the wane, and then evolves with a new breath triggered by the album “Rock’n’roll attitude”.

### 3.4. Julio Iglesias

Julio Iglesias is a Spanish songwriter and singer. Iglesias’ career started in 1968 and has had plenty of commercial success and artistic recognition, with more than 300 million records sold, about 5000 concerts for many millions of people, and dozens of awards worldwide. Iglesias is the most celebrated Latin music artist and one of the top 10 best-selling artists of all times.

Herein, we consider a total of 629 musical works included in 59 albums released in the period 1969–2007.

**Figure 11:**
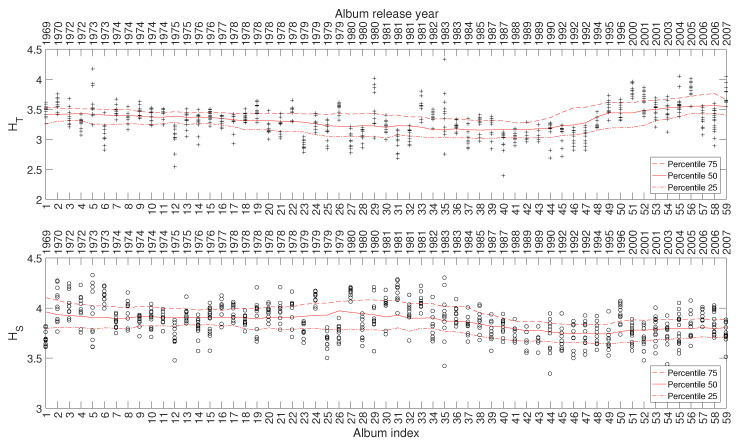
The complexity indices $H_{T}$ and $H_{S}$ versus i=1,⋯,59 for Julio Iglesias’ music.

Figure 11 depicts the 25, 50, and 75 percentiles of $H_{T}$ and $H_{S}$ versus i=1,⋯,59, showing a relationship between the evolution of the indices and the different periods of the artist’s career. Such a relationship also emerges in other complexity indices, but their corresponding charts are omitted here for the sake of parsimony.

**Figure 12:**
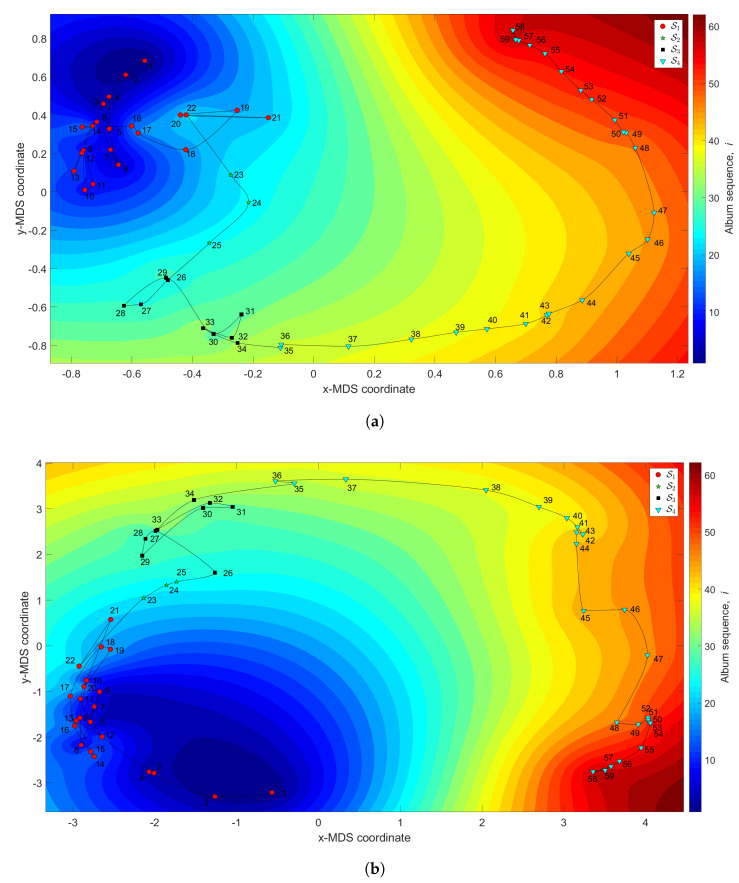
The MDS maps for Julio Iglesias’s music. The (x,y) coordinates are generated by means of the MDS with q=2 and the matrices: (**a**) $\Delta_{A}$; (**b**) $\Delta_{C}$. The *z* coordinate represents the albums’ sequence, *i*, interpolated with RBI.

Figure 12 depicts the 3-dimensional map generated with q=2 and dissimilarity matrices $\Delta_{A}$ and $\Delta_{C}$, with the *z* coordinate denoting the albums’ sequence, *i*, interpolated with RBI.

We verify the emergence of four clusters. The first, $S_{1}$ (i.e., albums 1≤i≤22), coincides with the height of Iglesias’ success during the 1970s and 1980s of the twentieth century. In this period we observe that the complexity evolves as a tangle confined to a small region in the plane. For $S_{2}$ (23≤i≤25), the complexity develops towards a new point, thanks to a few albums released in 1979. In the cluster $S_{3}$ (26≤i≤34), the trajectory reaches another small tangle, corresponding to musical albums recorded between 1979 and 1982. This period precedes a fourth cluster, $S_{4}$ (35≤i≤59), characterized by a large route and coinciding with albums released from 1983 up to 2007. Within this period, Iglesias started releasing many records tailored to suit American fans, including duets with some American stars. He then returned to the his Latin audience, including strengthening the relationship with his French followers, by releasing some French-language albums. In this period, Iglesias won the World Music Award and enjoyed major commercial success in Spain.

## 4. Conclusions

We adopted complexity, dimensionality-reduction, and visualization techniques for studying the music of several contemporary artists. The musical works were converted into digital format and represented in `mono’. The TS were assessed by means of eight distinct complexity indices. The 8-dimensional measurements were reduced to 2- and 3-dimensional by means of the MDS technique. The results revealed that the evolution of complexity is correlated with the artists’ musical careers. We conclude that the proposed indices represent reliable and assertive tools for assessing musical complexity.
